# Cross-Cultural Adaptation and Psychometric Validation of the Serbian Version of the Back Beliefs Questionnaire in Patients with Chronic Low Back Pain

**DOI:** 10.3390/medicina62061174

**Published:** 2026-06-17

**Authors:** Ivana Minaković, Tanja Janković, Mirjana Smuđa, Bela Kolarš, Monika Šili, Vesna Mijatović Jovin, Jelena Zvekić-Svorcan

**Affiliations:** 1 Faculty of Medicine, University of Novi Sad, 21000 Novi Sad, Serbia; tanja.jankovic@mf.uns.ac.rs (T.J.); bela.kolars@mf.uns.ac.rs (B.K.); 903012d23@mf.uns.ac.rs (M.Š.); jelena.zvekic-svorcan@mf.uns.ac.rs (J.Z.-S.); 2 Health Center “Novi Sad”, 21000 Novi Sad, Serbia; 3 Special Hospital for Rheumatic Diseases Novi Sad, 21000 Novi Sad, Serbia; 4 Department of Higher Medical School, The Academy of Applied Studies Belgrade, 11000 Belgrade, Serbia; mirjana.smudja@assb.edu.rs; 5 Department of Pharmacology, Toxicology and Clinical Pharmacology, Faculty of Medicine, University of Novi Sad, 21000 Novi Sad, Serbia; vesna.mijatovic-jovin@mf.uns.ac.rs

**Keywords:** back beliefs questionnaire, pain beliefs, low back pain, psychometric properties, validation, Serbian language

## Abstract

*Background and Objectives:* The Back Beliefs Questionnaire (BBQ) is a patient-reported outcome measure used to assess beliefs about back pain. This study aimed to cross-culturally adapt the BBQ into Serbian and evaluate the psychometric properties of the Serbian version (BBQ-Srb) in patients with chronic low back pain. *Materials and Methods:* A cross-sectional study involving cross-cultural adaptation and psychometric validation was conducted in 143 patients with chronic low back pain. The adaptation process included forward and backward translation, expert review, and pilot testing. Psychometric evaluation included assessment of floor and ceiling effects, internal consistency, test–retest reliability, measurement error, exploratory and confirmatory factor analyses, and construct validity testing using predefined hypotheses. Construct validity was examined through associations between BBQ-Srb scores and pain intensity, disability, pain catastrophizing, and work absenteeism. *Results:* The BBQ-Srb showed acceptable internal consistency, with Cronbach’s alpha of 0.728 and McDonald’s omega of 0.735. Total-score analyses were based on the preliminary exploratory 8-item BBQ-Srb version excluding BBQ13, whereas floor and ceiling effects were examined for the original 9-item scored BBQ-Srb version. Test–retest reliability was excellent (ICC = 0.916). Exploratory factor analysis suggested a predominantly one-factor structure, but the explained variance was modest. Confirmatory factor analysis of the 8-item version provided only partial support for unidimensionality, with marginal model fit and a low average variance extracted. The 8-item BBQ-Srb total score showed significant negative correlations with pain intensity, disability, and pain catastrophizing, confirming three of four predefined hypotheses. *Conclusions:* The BBQ-Srb demonstrated acceptable reliability and preliminary evidence of construct validity as a Serbian patient-reported outcome measure for assessing beliefs about back pain. However, structural validity was only partially supported, and the exploratory 8-item structure requires confirmation in larger, independent, and more diverse Serbian-speaking samples.

## 1. Introduction

Low back pain (LBP) is a highly prevalent clinical condition and is most often classified as non-specific, meaning that the precise anatomical origin of pain cannot be clearly identified [1,2]. Pain perception and pain-related disability result from the interplay of biological, psychological, and social factors [2,3,4]. Within this biopsychosocial framework, patients’ beliefs about LBP are clinically important because they may influence disability, functional outcomes, treatment adherence, recovery expectations, and health-related behavior [5,6]. Maladaptive beliefs, fear-avoidance behavior, and negative expectations about recovery have been associated with persistent pain, increased disability, greater healthcare utilization, work-related absenteeism, and poorer long-term outcomes [7,8,9,10]. Accurate assessment of back pain beliefs is therefore important in both clinical practice and research.

The Back Beliefs Questionnaire (BBQ) is a brief self-report instrument used to assess patients’ beliefs about low back problems, particularly the extent to which they expect back pain to have negative long-term consequences for everyday activities and work [11,12]. This construct is related to, but distinct from, constructs assessed by other commonly used instruments. The Pain Catastrophizing Scale (PCS) focuses on catastrophic thinking about pain [13], the Oswestry Disability Index (ODI) assesses pain-related disability [14], the Fear-Avoidance Beliefs Questionnaire (FABQ) assesses fear-avoidance beliefs related to physical activity and work [15], and the Pain Self-Efficacy Questionnaire (PSEQ) assesses confidence in functioning despite pain [16]. Therefore, the BBQ may complement these measures by identifying maladaptive beliefs relevant to patient education, activity recommendations, and rehabilitation planning [11,17].

In Serbian-speaking patients, the availability of validated instruments for assessing pain-related psychosocial constructs remains limited. To our knowledge, no published psychometric validation of a Serbian version of the BBQ was available before the present study. Among related instruments, the Fear Avoidance Components Scale has been validated in Serbian [18]. However, measurement properties are context-dependent and may vary across clinical populations [19]. Moreover, although the Fear Avoidance Components Scale assesses fear-avoidance components related to pain, it is not specifically designed to assess back pain beliefs in the way captured by the BBQ. In routine practice and research, back pain beliefs may therefore be addressed only indirectly, for example, through clinical interviews or translated instruments assessing related psychosocial constructs. However, for several commonly used related measures, published evidence of cross-cultural adaptation and psychometric validation in Serbian remains limited or unavailable. This gap limits the systematic assessment of back pain beliefs in Serbian-speaking clinical and research settings.

Cultural, occupational, and healthcare contexts may shape how patients understand and respond to back pain. Beliefs about LBP can be influenced by previous pain experiences, health literacy, sociocultural background, work-related demands, expectations regarding sickness absence and return to work, and experiences with healthcare providers [20,21,22,23,24]. In the Serbian context, these considerations may be relevant when adapting the BBQ, because patients’ beliefs can be shaped by healthcare access, occupational demands, experiences with medical treatment and sick leave, and attitudes toward rest, activity, and chronic pain. Therefore, formal cross-cultural adaptation and psychometric validation are needed to ensure that the Serbian BBQ (BBQ-Srb) is conceptually equivalent, culturally appropriate, and psychometrically sound in Serbian-speaking patients.

Previous validation studies have generally supported the BBQ as a useful instrument for assessing back pain beliefs, but they have also shown variability in its psychometric properties across languages and cultural contexts. Reported internal consistency has typically been acceptable, while test–retest reliability has ranged from moderate to high across different adaptations [6,7,17,25,26,27]. Although the original instrument and several validation studies have supported a unidimensional structure, other adaptations have reported three- or four-factor solutions, suggesting that the factor structure may vary depending on the sample and cultural context [7,10,11,20,25,27,28,29,30]. Some studies have also identified problematic items or item-level floor/ceiling effects, although these effects have not been consistently reported across validations [20,25,27,30]. In addition, BBQ scores have generally shown expected negative correlations with pain intensity, disability, and catastrophizing, supporting construct validity by showing expected associations with related constructs. However, these associations are often weak to moderate because the BBQ assesses beliefs about the consequences of back pain rather than current symptom severity or disability [10,25,26,27,30].

Therefore, this study aimed to translate and culturally adapt the BBQ into Serbian and to evaluate the psychometric properties of the BBQ-Srb in Serbian-speaking patients with LBP. Establishing a culturally adapted Serbian version of the BBQ with initial psychometric evidence may support the standardized assessment of back pain beliefs and contribute to improved clinical evaluation and research in this population.

## 2. Materials and Methods

### 2.1. Population and Study Context

This was a cross-sectional study involving the cross-cultural adaptation and psychometric validation of a patient-reported outcome measure, the BBQ-Srb. The study included patients treated for chronic LBP, defined as pain lasting for at least three months, at the Special Hospital for Rheumatic Diseases, Novi Sad, Serbia. Participants were recruited from October 2025 to February 2026. Ethical approval was obtained from the Ethics Committee of the Special Hospital for Rheumatic Diseases, Novi Sad, Serbia (approval number: 14/01-2/1-25), and all participants provided written informed consent prior to enrollment.

Patients were consecutively recruited if they met the predefined inclusion and exclusion criteria. Inclusion criteria comprised cognitively able participants of both sexes, aged 40–65 years, with pain in the lumbosacral region of the spine and a pain intensity score of ≥5 on the Numeric Rating Scale (NRS). The age range of 40–65 years was selected to obtain a relatively homogeneous and clinically relevant validation sample. This range included patients in middle and late working age, for whom chronic LBP may have important functional, occupational, and psychosocial consequences. Restricting the age range also helped reduce heterogeneity related to younger adults, whose occupational and lifestyle profiles may differ substantially, and older adults, in whom comorbidities, degenerative conditions, and age-related functional limitations may have a stronger influence on pain-related beliefs and disability. The requirement of moderate to severe pain intensity, defined as an NRS score ≥ 5, was used to ensure that participants had clinically meaningful symptoms at the time of assessment.

Exclusion criteria included insufficient proficiency in Serbian or inability to complete the questionnaire independently, as well as the presence of rheumatic diseases, previous spinal injuries or fractures, or a history of spinal surgery.

A total of 166 eligible patients were initially recruited. Of these, 23 patients were excluded from the final analysis because they did not complete the retest assessment or had missing data to an extent that made their responses unsuitable for analysis. Therefore, the final validation sample consisted of 143 participants.

The sample size was determined according to commonly used recommendations for the psychometric evaluation and validation of measurement instruments, including participant-to-item ratios of at least 5–10 participants per item [31]. Although the BBQ contains 14 items in total, only 9 items contribute to the final score, whereas 5 items are distractor items. Therefore, the minimum sample size for the main psychometric analyses was estimated based on the 9 scored items, resulting in N = 90 participants. The final validation sample included 143 participants, corresponding to approximately 16 participants per scored item and exceeding this minimum requirement. Even when all 14 questionnaire items are considered, the sample corresponds to approximately 10 participants per item.

All participants completed the BBQ-Srb, ODI, and PCS questionnaires. Pain intensity was assessed using the NRS. During the visit, demographic and clinical data were collected, including age, marital status, cohabitation, education, occupation, employment status, physical workload, and frequency of work absence. Pain was classified as localized back pain or back pain radiating to the legs.

The study was conducted in two stages. In the first stage, the BBQ was translated into Serbian and culturally adapted. In the second stage, the psychometric properties of the Serbian version were evaluated.

### 2.2. Process of Translation and Cultural Adaptation

The translation and cross-cultural adaptation process followed established international guidelines [32], while psychometric properties were reported and categorized in accordance with the COSMIN recommendations [33].

The original English version of the BBQ was translated into Serbian after permission had been obtained from Prof. A. K. Burton, one of the original developers of the instrument, to adapt the questionnaire and evaluate its reliability and validity in Serbian-speaking patients.

The translation and cross-cultural adaptation process followed standard procedures for questionnaire adaptation. First, two independent forward translations from English into Serbian were performed by native Serbian speakers who were highly proficient in English. The two translated versions were then compared and harmonized into a single preliminary Serbian version. Subsequently, two independent professional translators proficient in both Serbian and English, who were blinded to the original English version of the questionnaire, performed back-translation into English.

An expert committee was then formed to review all translated versions, resolve discrepancies, and ensure conceptual and linguistic equivalence between the original and Serbian versions. The committee included clinicians, translators, and a psychologist with expertise in statistics and psychometrics, who contributed as the methodology expert. During this process, semantic, idiomatic, experiential, and conceptual equivalence were assessed. The committee also qualitatively evaluated content-related aspects of the Serbian version, including item relevance, cultural appropriateness, clarity, and whether the translated items adequately reflected the intended content of the original BBQ. However, no separate formal quantitative assessment of content validity, such as item rating scales or a content validity index, was performed.

After consensus had been reached regarding terminology, wording, and formatting, a pre-final Serbian version of the BBQ was prepared. This version was pilot-tested with a convenience sample of 15 female patients with LBP using cognitive debriefing questions. The purpose of this phase was to evaluate patients’ comprehension, interpretability, and perceived clarity of the items. Based on participants’ feedback, only two minimal wording clarifications were made in the final Serbian version. In item 1, the initial Serbian wording proposed by the translators implied a treatment that would completely cure back trouble; therefore, it was revised to convey the meaning of “real treatment” as “truly effective treatment,” which better preserved the conceptual meaning of the original item. In item 12, the phrase “in the back” was added at the end of the Serbian sentence to avoid interpreting “weakness” as general bodily weakness and to clarify that the item referred specifically to back-related weakness. These clarifications were made to preserve conceptual equivalence with the original items rather than to alter their meaning. No substantial changes to item meaning were introduced. The final BBQ-Srb was reviewed and approved by Prof. A. K. Burton.

The BBQ-Srb was administered at two time points: at study enrollment and again after seven days, to assess test–retest reliability. During this interval, no treatment was provided, and participants were instructed to continue their usual behaviors related to LBP.

### 2.3. Clinical Assessment Tools

#### 2.3.1. Back Beliefs Questionnaire (BBQ)

The BBQ was designed to assess beliefs about the consequences of low back problems. The instrument consists of a single scale derived from nine core items, supplemented by five additional statements serving as distractors. Responses to each item are recorded on a 5-point Likert scale, ranging from 1 (strongly disagree) to 5 (strongly agree). Total scores are obtained by reverse scoring and summing the nine core items, yielding a possible range from 9 to 45. Lower total scores reflect more negative or pessimistic beliefs regarding LBP [11].

In the present Serbian validation sample, BBQ13 was excluded from the total-score calculation, resulting in a possible score range of 8–40.

#### 2.3.2. Oswestry Disability Index (ODI) Questionnaire

The ODI is a widely used outcome measure developed to evaluate functional limitations in activities of daily living related to LBP. The instrument comprises ten domains, each rated on a 6-point scale ranging from 0 to 5, with higher scores indicating greater levels of disability. The overall index score is calculated by summing the item scores, dividing by the maximum possible score, and expressing the result as a percentage. The ODI is regarded as the gold standard for assessing functional outcomes associated with LBP and is commonly employed to quantify long-term functional disability in affected individuals [14]. The Serbian (Serbia) language version of the ODI, version 2.1a (Version of 12 February 2016), was administered in paper-and-pencil format with permission from MAPI Research Trust.

#### 2.3.3. Pain Catastrophizing Scale (PCS)

The PCS comprises 13 items rated by participants on a 5-point Likert scale ranging from 0 (“not at all”) to 4 (“all the time”), reflecting the frequency with which specific thoughts or feelings are experienced in pain-related situations. The total PCS score is obtained by summing responses across all items, with higher scores indicating greater levels of pain catastrophizing. The scale includes three subscales: rumination, magnification, and helplessness [13]. The Serbian (Serbia) language version of the PCS (Version of 5 May 2011) was administered in paper-and-pencil format with permission from MAPI Research Trust.

### 2.4. Data Analysis

For the purpose of evaluating the psychometric properties of the BBQ-Srb, structural validity, construct validity by hypothesis testing, and reliability were assessed. Content-related aspects were addressed qualitatively during the translation and cross-cultural adaptation process, as described above. Psychometric analyses were initially conducted for the original 9 scored BBQ items. Because item BBQ13 showed poor psychometric performance, an exploratory 8-item BBQ-Srb model excluding BBQ13 was subsequently evaluated for structural validity. After exclusion of BBQ13, the preliminary exploratory 8-item BBQ-Srb total score was used in all subsequent total-score analyses, including descriptive statistics, internal consistency, test–retest reliability, SEM, SDC, construct-validity correlations, subgroup analyses, and logistic regression. Floor and ceiling effects were examined for the original 9-item scored BBQ-Srb version to document item-level response distributions, including the performance of BBQ13. Structural validity was examined using exploratory factor analysis (EFA) and confirmatory factor analysis (CFA). Both EFA and CFA were conducted on the same final validation sample (N = 143), and this issue is acknowledged as a limitation.

Construct validity by hypothesis testing was evaluated by testing a priori hypotheses regarding the expected direction and magnitude of associations between BBQ-Srb scores and theoretically related constructs. Specifically, we hypothesized that the preliminary exploratory 8-item BBQ-Srb total score would show weak-to-moderate negative correlations with pain catastrophizing, functional disability, and pain intensity, as measured by the PCS, ODI, and NRS, respectively. We also hypothesized that lower BBQ-Srb scores would be associated with greater odds of work absenteeism.

These hypotheses were tested using Pearson’s correlation coefficients for the associations between BBQ-Srb scores and PCS, ODI, and NRS scores, as the BBQ-Srb total score was normally distributed according to the Shapiro–Wilk test. The association between BBQ-Srb scores and work absenteeism was examined using binary logistic regression. Differences in BBQ-Srb scores across sociodemographic, clinical, and work-related characteristics were examined using one-way analysis of variance (ANOVA) and independent-samples *t*-tests, as appropriate. To reduce the risk of type I error due to multiple subgroup comparisons, the Benjamini–Hochberg false discovery rate (FDR) procedure was applied to adjust *p*-values. Effect sizes were reported as eta-squared (η^2^).

The reliability of the instrument was assessed in terms of internal consistency and test–retest stability. Internal consistency was evaluated using Cronbach’s α and McDonald’s ω, while test–retest stability was assessed using the intraclass correlation coefficient (ICC). Measurement precision and agreement were additionally assessed using the standard error of measurement (SEM), the smallest detectable change (SDC), and Bland–Altman analysis.

Descriptive statistics, including mean, standard deviation, minimum, and maximum values, were calculated, along with indicators of distribution shape, including skewness and kurtosis. For categorical variables, frequency and percentage distributions were presented in frequency tables. Data analysis was performed using IBM SPSS Statistics for Windows, Version 27.0 (IBM Corp., Armonk, NY, USA), and IBM SPSS Amos for Windows, Version 25.0 (IBM Corp., Armonk, NY, USA).

## 3. Results

### 3.1. Participant Characteristics

Within the examined sample (N = 143), men accounted for 30.8% of participants. Regarding age distribution, 23.1% of respondents were between 40 and 50 years old, 45.5% were between 51 and 60 years old, and 31.5% were between 61 and 65 years old.

In terms of body mass index (BMI), 27.9% of participants had normal body weight, 51.9% were classified as overweight, and 20.2% as obese. With respect to marital status, 70.6% of respondents were married, 8.4% were single, 9.1% were divorced or separated, and 11.9% were widowed.

Overall, 40.1% of participants reported living in a cohabiting arrangement. Most respondents had completed high school as their highest level of education (62.9%). General participant characteristics and descriptive scale scores are presented in Table 1. Inferential statistics for the associations between participants’ general characteristics and BBQ-Srb, NRS, ODI, and PCS scores are presented in Table 2.

### 3.2. Participants’ Occupational and Pain Profile

The occupational structure of the sample showed that 21.0% of participants were associate professionals and technicians, 26.6% were service and sales workers, 15.4% were clerical workers, and 9.8% were professionals, while 27.3% were classified in the “other” category. Most respondents had permanent employment contracts (67.1%), and slightly more than half (54.2%) reported that their job was aligned with their qualifications.

Regarding physical workload, 25.9% of participants predominantly performed sedentary work, 33.6% stood or walked without carrying loads, 26.6% engaged in more intensive movement with occasional lifting, and 14.0% performed heavy physical labor.

With respect to LBP, 65.5% of respondents reported experiencing pain both at rest and during activity, 29.6% reported pain only during activity, and 4.9% reported pain only at rest. Overall, 21.7% of participants reported pain localized exclusively in the lumbar region, 51.0% indicated that pain occasionally radiated down the leg, and 27.3% reported constant radiating pain. Symptoms occurred once per year in 8.7% of respondents, twice per year in 26.9%, and three or more times per year in 64.4%. Because of these symptoms, 33.7% of participants did not take sick leave, 37.5% were occasionally absent from work for a few days, 21.2% were frequently absent for several weeks, and 7.7% reported prolonged absences lasting several months.

No statistically significant differences in the preliminary exploratory 8-item BBQ-Srb total score were observed across the examined occupational and pain-related characteristics. Although participants who reported pain both at rest and during activity had lower 8-item BBQ-Srb total scores (M = 20.55, SD = 5.52) than those who experienced pain only during activity (M = 23.02, SD = 6.53) or only at rest (M = 22.71, SD = 6.68), this difference did not reach statistical significance (*p* = 0.306). Similarly, the 8-item BBQ-Srb total score did not differ significantly according to work absenteeism (*p* = 0.366), duration of absenteeism (*p* = 0.306), or frequency of absenteeism (*p* = 0.366). Detailed data are provided in Supplementary Tables S1 and S2.

In the unadjusted model, the 8-item BBQ-Srb total score was not statistically significantly associated with work absenteeism (OR = 1.034; 95% CI: 0.967–1.106). However, after adjustment for sex, age, disability level (ODI), pain catastrophizing (PCS), pain intensity (NRS), employment status, and physically demanding work, the 8-item BBQ-Srb total score showed a statistically significant association with work absenteeism (OR = 1.150; 95% CI: 1.040–1.270; *p* < 0.01). This association was in the opposite direction to the predefined hypothesis, indicating that higher 8-item BBQ-Srb total scores were associated with greater odds of work absenteeism in the adjusted model.

Among the included covariates, ODI also emerged as a statistically significant predictor, with higher levels of disability associated with a greater likelihood of the outcome (OR = 1.081; 95% CI: 1.033–1.132; *p* < 0.01). Compared with participants who reported physically demanding work as the reference category, those who mostly performed sedentary work had a significantly lower likelihood of the outcome (OR = 0.132; 95% CI: 0.021–0.805; *p* < 0.05), corresponding to an approximately 86% lower likelihood of work absenteeism. The other workload categories did not reach statistical significance. The results are presented in Table 3.

### 3.3. Cross-Cultural Validation of the Back Beliefs Questionnaire (BBQ)

Floor and ceiling effects for the original 9-item BBQ-Srb total score and original scored items were examined at both administrations using the commonly applied threshold of >15%. The results are presented in Table 4.

No floor effect was observed for the original 9-item BBQ-Srb total score at either administration (0% at both time points). The total-score ceiling effect was absent at the first administration (0%) and negligible at the second administration (0.7%), indicating that the original 9-item BBQ-Srb total score was not limited by clustering at either end of the possible score range. At the item level, floor effects were more frequent than ceiling effects. The most pronounced floor effects were observed for BBQ13 (56.6% at the first administration and 50.3% at the second administration) and BBQ14 (42.7% and 39.9%, respectively). BBQ13 also showed the lowest mean scores and limited endorsement of the highest response category (2.1% and 1.4%), supporting its poorer item-level performance. Ceiling effects were mainly observed for BBQ1 and BBQ8, with BBQ8 showing the highest ceiling percentages at both administrations (40.6% and 35.7%). These findings indicate that, although the original 9-item BBQ-Srb total score did not show meaningful floor or ceiling effects, several individual items, particularly BBQ13, showed marked response clustering that should be considered when interpreting item-level psychometric performance.

The results presented in Table 5 summarize the reliability and structural validity findings for the preliminary exploratory 8-item BBQ-Srb version. Item–total correlations (I–TC) ranged from 0.213 to 0.559. Internal consistency analysis of the initial 9-item scored BBQ-Srb version showed a Cronbach’s alpha of 0.725 (95% CI: 0.652–0.788). After exclusion of BBQ13, Cronbach’s alpha for the preliminary exploratory 8-item BBQ-Srb version was 0.728 (95% CI: 0.655–0.790), and this value is reported in Table 5. Test–retest reliability, assessed using the ICC based on absolute agreement, was high both at the item level (ICC = 0.68–0.92) and for the 8-item total score (ICC = 0.916; 95% CI: 0.883–0.940), indicating good temporal stability of the preliminary exploratory 8-item BBQ-Srb total score.

McDonald’s omega coefficient for the preliminary exploratory 8-item BBQ-Srb scale was ω = 0.735, indicating acceptable internal consistency. The SEM was 1.69, while the SDC was 4.68. Bland–Altman analysis showed a mean difference of −0.41 between test and retest scores, with 95% limits of agreement ranging from −4.98 to 4.16, indicating reasonable agreement between repeated measurements.

The Kaiser–Meyer–Olkin (KMO) measure of sampling adequacy was 0.737, and Bartlett’s test of sphericity was significant (χ^2^ = 232.786; df = 36; *p* < 0.001), indicating that the data were suitable for EFA. EFA using principal axis factoring suggested a predominantly unidimensional structure of the instrument, identifying one dominant factor with an eigenvalue of 2.870, which explained 32.5% of the total variance. Although the second factor demonstrated an eigenvalue slightly above 1 (1.150), inspection of the scree plot supported retention of a predominantly unidimensional solution. Parallel analysis was not performed. Factor retention was therefore based on eigenvalues, inspection of the scree plot, item factor loadings, and interpretability of the solution. Item factor loadings ranged from 0.344 to 0.707, while item communalities ranged from 0.118 to 0.500. However, item BBQ13 (“Back trouble must be rested”) demonstrated a very low factor loading and weak association with the total scale score. Therefore, an exploratory 8-item BBQ-Srb model excluding BBQ13 was subsequently evaluated.

CFA of the initial single-factor model, conducted using the Maximum Likelihood estimator, revealed a marginally acceptable model fit. Due to the very low factor loading of item BBQ13 (β = 0.18), which was below the recommended threshold of 0.30, CFA was repeated for the exploratory 8-item BBQ-Srb model excluding this item. In this exploratory 8-item model, standardized factor loadings ranged from 0.321 to 0.722, all of which were statistically significant (*p* < 0.05), indicating that each item contributed meaningfully to the latent construct.

The model fit indices suggested a marginal fit to the empirical data (χ^2^(20) = 41.82; χ^2^/df = 2.09; CFI = 0.889; TLI = 0.844; RMSEA = 0.088; 90% CI: 0.050–0.125). Composite reliability was acceptable (CR = 0.74), whereas the average variance extracted was below the recommended threshold (AVE = 0.28), indicating that the latent factor explained a limited proportion of item variance. The SRMR index was 0.143, indicating a moderate discrepancy between the model and the empirical data.

**Figure 1:**
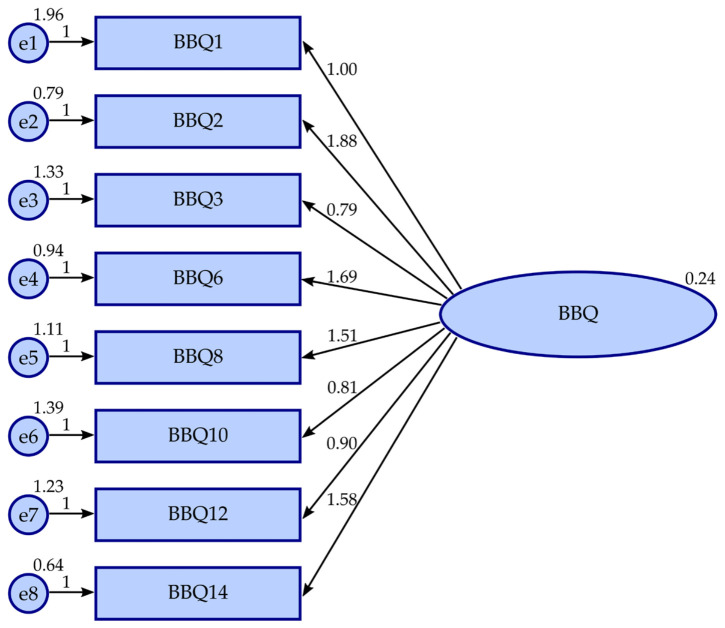
Confirmatory factor analysis model of the preliminary exploratory 8-item BBQ-Srb scale. Note. Rectangles indicate observed variables. The ellipse represents the latent variable, and small circles denote error terms. Unstandardized regression coefficients (B) are presented in the path diagram.

Unstandardized coefficients (B) ranged from 0.79 to 1.58. The highest loading was observed for item BBQ14 (B = 1.58), while the lowest loading was found for item BBQ3 (B = 0.79), as shown in Figure 1.

Construct validity was assessed using a hypothesis-testing approach. Four predefined hypotheses were formulated regarding the expected associations between BBQ-Srb scores and pain intensity, disability, pain catastrophizing, and work absenteeism. Three of the four hypotheses were confirmed. The preliminary exploratory 8-item BBQ-Srb total score showed statistically significant negative correlations with pain intensity measured by NRS (r = −0.256, *p* = 0.002), disability measured by ODI (r = −0.284, *p* = 0.001), and pain catastrophizing measured by PCS (r = −0.403, *p* < 0.001). The direction of these correlations was consistent with the predefined hypotheses, whereas the hypothesis regarding work absenteeism was not confirmed (Table 6).

**Figure 2:**
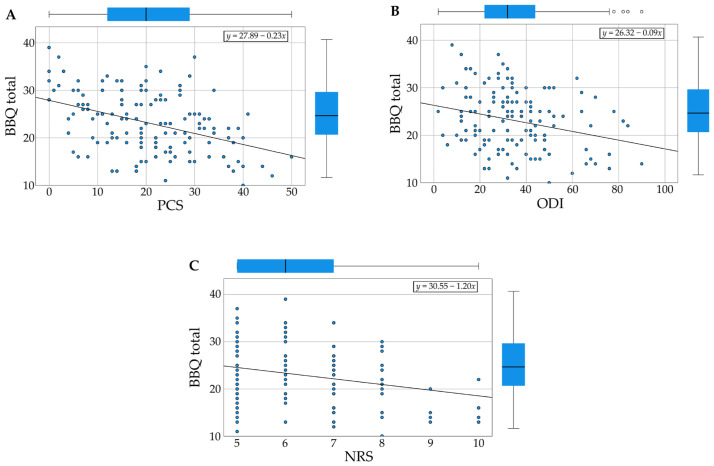
Correlations between the preliminary exploratory 8-item BBQ-Srb total score and PCS, ODI, and NRS. (**A**) Correlation between the BBQ-Srb and PCS scores; (**B**) Correlation between the BBQ-Srb and ODI scores; (**C**) Correlation between the BBQ-Srb and NRS scores.

Correlations between the 8-item BBQ-Srb total score and NRS, PCS, and ODI are presented in Figure 2.

## 4. Discussion

This study focused on the translation and cross-cultural adaptation of the BBQ into Serbian, followed by an evaluation of its reliability and validity. The findings suggest that the BBQ-Srb demonstrates acceptable reliability and preliminary evidence of construct validity by hypothesis testing, whereas structural validity was only partially supported and requires further confirmation.

For clarity, all total-score analyses reported in this study refer to the preliminary exploratory 8-item BBQ-Srb version excluding BBQ13. Regarding reliability, item–total correlation (I-TC) values ranged from 0.213 to 0.559. Internal consistency analysis showed that Cronbach’s alpha for the preliminary exploratory 8-item BBQ-Srb was 0.728, while the values for individual items (CA-iid) were also within acceptable limits. The results obtained are in line with prior research, where Cronbach’s alpha values varied between 0.67 and 0.82 [6,7,17,25,26,27]. Test–retest reliability, assessed using the ICC, was high for both individual items and the 8-item total score (ICC = 0.916; 95% CI: 0.883–0.940). Similarly, high ICC values have been reported in the original English version (ICC = 0.87) [11], as well as in studies that performed cross-cultural validation of the BBQ [7,25,30,34]. In contrast, lower values have been reported in some versions, such as the Norwegian (ICC = 0.71), Italian (ICC = 0.72), and French (ICC = 0.64) versions [10,17,27].

Previous studies have examined the psychometric properties of the BBQ and reported variability in factor loadings across cultural adaptations. Most validations, including the original questionnaire, have supported a unidimensional structure [11,27,28]. However, several studies have reported three-factor [7,20,25,29] or four-factor solutions [10,30]. Importantly, even in studies reporting multidimensional solutions, the eight or nine scored items often loaded primarily on the first factor [7,25,29]. Similarly, in the Yoruba version, although a three-factor structure was identified, six of the scored items loaded on a single factor [20]. In the present study, the adequacy of the data for EFA was supported by the KMO value (0.737) and a significant Bartlett’s test of sphericity. This KMO value was comparable to those reported by Ibrahim et al. [30] (0.754) and Mbada et al. [20] for the Yoruba version (0.742), indicating similar sampling adequacy across these validation studies. EFA using principal axis factoring indicated a predominantly one-factor solution, with the first factor explaining 32.5% of the variance and factor loadings ranging from 0.344 to 0.707. Although the explained variance was modest and the second factor had an eigenvalue slightly above 1, the scree plot, item loading pattern, and interpretability of the solution supported retention of a single dominant factor. However, because parallel analysis was not performed and the explained variance was modest, this finding should be interpreted as preliminary support for a predominantly unidimensional structure rather than as strong confirmation of unidimensionality. Overall, these results are consistent with the view that the preliminary exploratory 8-item BBQ-Srb version primarily captures a general construct of beliefs about back problems, while differences across studies may reflect cultural and linguistic adaptation, sample characteristics, item functioning, extraction methods, rotation procedures, and factor-retention criteria.

In our study, BBQ13 (“Back trouble must be rested”) showed consistently weak psychometric performance. Because of this finding, the preliminary exploratory 8-item BBQ-Srb model excluding BBQ13 was evaluated. The item had a very low factor loading in the EFA (0.108), a low standardized factor loading in the CFA (β = 0.18), below the commonly used minimum threshold of 0.30, and an almost zero corrected item–total correlation (−0.001). Although removal of BBQ13 produced only a minimal increase in Cronbach’s α, from 0.725 to 0.728, the convergence of evidence from item–total correlation, EFA, and CFA indicated that this item did not contribute adequately to the homogeneity of the measurement model. The subsequent CFA of the Serbian version provided only cautious and partial support for a predominantly unidimensional 8-item BBQ-Srb structure, as the overall model fit was marginal rather than optimal (χ^2^/df = 2.09; CFI = 0.889; TLI = 0.844; RMSEA = 0.088, 90% CI: 0.050–0.125; SRMR = 0.143). Standardized factor loadings for the retained items were statistically significant and ranged from 0.321 to 0.722, indicating that each retained item contributed to the latent construct. Composite reliability was acceptable (CR = 0.74), whereas the low AVE value (0.28) indicated that the latent factor explained a limited proportion of item variance. This finding weakens the evidence for a strong unidimensional measurement model and suggests that the retained items did not share a sufficiently high proportion of common variance. However, these results should be interpreted together with the hypothesis-testing findings, which showed theoretically consistent associations with pain intensity, disability, and pain catastrophizing. Overall, the findings provide preliminary but not conclusive support for the structural validity and construct validity by hypothesis testing of the 8-item BBQ-Srb.

One possible explanation for the weak performance of BBQ13 is that this item may be particularly sensitive to contextual interpretations of LBP management. Direct Serbian prevalence data on the belief that LBP requires rest are limited. However, the Serbian national guideline for lumbar syndrome explicitly recommends that clinicians advise patients to avoid rest and resume usual activities as soon as possible [35]. At the same time, Serbian clinical sources have documented fear-avoidance beliefs, reduced activity, and avoidance of physical and work activities among patients with LBP [36,37], while a local emergency-medicine audit reported that rest was among the most common pieces of advice given to patients with lumbar pain syndrome in prehospital care [38]. Therefore, BBQ13 may not have functioned only as a general indicator of pessimistic back beliefs, but may also have captured a context-dependent and potentially ambiguously interpreted belief about rest and activity during LBP. With regard to item reduction and factor-structure instability, these findings can be compared with the German validation study, in which CFA supported an 8-item model after item 1 had been excluded. In that study, EFA initially yielded a three-factor solution, although the original scored items, except item 1, loaded primarily on the first factor [29]. Overall, while the present findings provide preliminary support for an exploratory 8-item Serbian version, they also indicate that the measurement model should be further examined and confirmed in future studies.

It is important to note that most validation studies of the BBQ did not report item-level floor or ceiling effects. When assessed, these effects were usually defined as present if more than 15% of participants achieved the lowest or highest possible total score [7,10,20,39,40]. However, item-level distributions were rarely reported; both in studies that performed cross-cultural validation of the BBQ and in the original development study, detailed item response counts or per-item percentages were generally not provided [6,10,11,26,40]. Consistent with most previous studies, our findings indicated that, at the original 9-item total-score level, no participants reached either the minimum or maximum possible score, suggesting that the BBQ-Srb effectively captures the full spectrum of back-related beliefs in our sample.

Among the studies that validated the BBQ, only a few (French, Hausa and Turkish) reported item-level floor or ceiling data [25,27,30]. Dupeyron et al. noted that only item 8 exhibited a substantial floor effect (60%), whereas ceiling effects were observed for items 2, 3 and 6 [27]. In the study by Karaman and Küçükakkaş [25], a ceiling effect was reported for items 3, 6, 10, 13 and 14, while a floor effect was observed for items 1, 2 and 8. This pattern was opposite to that observed in the present study, possibly reflecting differences in scoring direction, sample characteristics, or response patterns. The authors attributed these findings to the relatively small sample size and the use of a 5-point Likert scale. Ibrahim et al. [30] reported floor effects on eight items (2, 5, 7, 9, 11, 12, 13 and 14), which aligns closely with our results, where floor effects were observed for most items. In our study, the most pronounced floor effect was observed for item 13 (56.6%). These floor effects are consistent with our CFA results, as item 13 also demonstrated a particularly low factor loading (β = 0.18).

The preliminary exploratory 8-item BBQ-Srb demonstrated acceptable absolute reliability and agreement between repeated measurements, with an SEM of 1.69 and an SDC of 4.68. These values indicate relatively low measurement error and are consistent with the high test–retest reliability observed in the present study. They are broadly comparable with those reported by Ibrahim et al. [30], who found SEM and MDC95 values of 1.9 and 5.2, respectively, together with similar Bland–Altman limits of agreement. In contrast, the preliminary exploratory 8-item BBQ-Srb version showed more favorable measurement error indices than the Brazilian version reported by Teixeira et al. [34] and the Norwegian version reported by Tingulstad et al. [17], both of which showed higher SEM and SDC/MDC values and wider Bland–Altman limits of agreement. Differences in measurement error indices across validation studies should be interpreted cautiously, given differences in sample characteristics, adapted scale versions, scoring ranges, and analytic approaches.

The results of this study indicated significant negative correlations between the preliminary exploratory 8-item BBQ-Srb version and the NRS (r = −0.256, *p* = 0.002), ODI (r = −0.284, *p* = 0.001), and PCS (r = −0.403, *p* < 0.001), suggesting that higher BBQ scores, reflecting more adaptive beliefs, are associated with lower levels of pain, disability, and catastrophizing. Similar patterns have been observed in several cross-cultural validation studies: the Italian version demonstrated a moderate negative correlation with PCS (ρ = −0.45) [10], the French version reported a comparable association (ρ ≈ −0.45) [27] and the Hausa (Nigerian) adaptation found a weaker negative correlation with PCS (ρ ≈ −0.20) [30]. Regarding disability, the BBQ–ODI correlation in the present study was consistent with those reported for other versions, including the Turkish (ρ = −0.42) [25], Hausa (ρ = −0.30 to −0.42) [30], and traditional Chinese (ρ = −0.341) adaptations [26]. This consistency supports construct validity by hypothesis testing, although the association should be interpreted as weak to moderate rather than strong. The results of the present study showed a weak negative correlation between the preliminary exploratory 8-item BBQ-Srb version and NRS, in line with previous studies reporting similar negative associations. This relationship was very weak in the Arabic (ρ = −0.106) and Norwegian (ρ = −0.14) versions, whereas a moderate negative correlation was observed in the Turkish version (ρ = −0.34) [2,7,25]. The correlation of the BBQ with pain and disability scales is considered potentially weak, as the BBQ primarily assesses an individual’s perceptions of obstacles that may arise in the future due to current or anticipated pain, rather than directly measuring the current level of pain or disability [25]. Overall, these findings suggest that the relationship between adaptive back beliefs and more favorable pain-related profiles is broadly consistent across international validation studies.

Results from previous research support that work-related fear-avoidance beliefs increase the risk of long-term sickness absence in workers with musculoskeletal pain [22,23], including patients with chronic LBP [24], emphasizing the need for interventions targeting these beliefs. These results align with the initial validation of the English BBQ, which demonstrated that the instrument could differentiate workers holding maladaptive beliefs associated with prolonged work absenteeism, highlighting its utility for capturing beliefs relevant to work-related outcomes [11]. However, in the present study, the predefined hypothesis regarding work absenteeism was not supported. Although BBQ-Srb scores were significantly associated with work absenteeism after adjustment for sex, age, disability, pain catastrophizing, pain intensity, employment status, and physical workload, the direction of the association was opposite to that expected: higher BBQ-Srb scores were associated with greater odds of work absenteeism. This unexpected finding should be interpreted cautiously and should not be taken as evidence that more adaptive back beliefs increase the risk of work absenteeism. Rather, it is consistent with systematic-review evidence indicating that the prospective relationship between back-pain beliefs and future pain-related outcomes was insufficiently established in the literature reviewed [41]. Future longitudinal studies using more detailed measures of sickness absence are needed to clarify the relationship between BBQ-Srb scores and work-related outcomes.

In this study, the preliminary exploratory 8-item BBQ-Srb total score did not differ significantly across general demographic characteristics, such as gender, age, BMI, marital status, or education. This aligns with previous research, where demographic factors were either weakly associated or explained only a small portion of the variance in BBQ scores [2,25,28,30]. On the other hand, the study validating the Hausa version of the BBQ reported associations between lower education and more pessimistic beliefs about back pain, which likely reflects the fact that 33% of participants in their study had non-formal education and 55.5% were illiterate [30]. More broadly, it is well recognized that beliefs about chronic LBP are shaped by cultural background, sociocultural environment, prior pain experiences and health literacy [20,21]. These factors may contribute to variations in the way patients perceive, report and respond to back pain across different populations.

From a clinical perspective, the preliminary exploratory BBQ-Srb version may have practical value as a brief measure for identifying patients with more maladaptive beliefs about back pain in Serbian-speaking clinical populations. In rehabilitation and occupational health settings, it may help clinicians recognize patients who may benefit from education-based interventions addressing misconceptions about back pain, fear of movement, avoidance of activity, and beliefs about the need for rest or protection. The questionnaire may also contribute to broader psychosocial assessment when used alongside measures of pain intensity, disability, catastrophizing, and work-related functioning. However, the BBQ-Srb should not yet be used as a standalone tool for guiding treatment decisions or evaluating clinically meaningful change. Further longitudinal studies are needed to confirm responsiveness, interpretability, and thresholds for meaningful change before such applications can be recommended.

This study has several limitations. First, participants were recruited from a single center, which may limit the generalizability of the findings. Although the inclusion criteria were intentionally defined to obtain a relatively homogeneous and clinically relevant validation sample of middle- and late-working-age patients with chronic LBP and pain intensity of NRS ≥ 5, this approach also limits the applicability of the findings to younger and older adults, patients with acute or subacute LBP, and individuals with milder pain intensity. Second, the sample size was justified using commonly applied participant-to-item recommendations for preliminary psychometric validation studies; however, a formal a priori sample size calculation was not performed. In addition, EFA and CFA were conducted in the same sample, and the CFA sample size was limited. Because CFA is ideally conducted in an independent validation sample, the structural validity findings should be interpreted as preliminary and require confirmation in a larger independent sample. Third, the preliminary exploratory 8-item BBQ-Srb model excluding item 13 was evaluated based on the poor psychometric performance of this item in the present sample, but this modified structure has not yet been externally validated. Because this model differs from the original 9-item scoring structure, direct comparisons with the original BBQ and other international BBQ versions should be made cautiously. Future studies should therefore examine whether this item structure can be replicated in independent samples. Fourth, although the translation and cross-cultural adaptation process included expert review and cognitive debriefing, formal content validity was not assessed using quantitative methods such as item rating scales or a content validity index. In addition, pilot testing was conducted only among women, which may have limited the assessment of item comprehensibility across the full target population. Fifth, responsiveness was not assessed because the study had a cross-sectional design and did not include longitudinal follow-up, an intervention, or an external criterion of clinical change. Although SEM and SDC provide information on measurement error and the magnitude of change that can be interpreted as real change beyond measurement error, they do not substitute for a formal responsiveness analysis. Future longitudinal studies should therefore evaluate the responsiveness of the BBQ-Srb and its ability to detect change over time. Finally, the subgroup and work-related analyses should be interpreted cautiously. Although correction for multiple comparisons was applied where appropriate and multivariable logistic regression was added for work absenteeism, these analyses were exploratory and were not the primary focus of the validation study.

## 5. Conclusions

The BBQ-Srb showed acceptable internal consistency and excellent test–retest reliability in this sample of patients with chronic LBP. Expected associations with pain intensity, disability, and pain catastrophizing provided preliminary support for construct validity. However, structural validity was only partially supported, and item 13 showed poor psychometric performance in the present sample. Therefore, the BBQ-Srb should currently be considered a preliminary Serbian version with initial validation evidence rather than a fully confirmed instrument. Further studies in larger, independent, and more diverse Serbian-speaking populations are needed to confirm the factor structure, externally validate the modified item structure, evaluate interpretability and thresholds for clinically meaningful change, and assess responsiveness in longitudinal designs.

## Figures and Tables

**Table 1:** General participant characteristics and descriptive scale scores.

Characteristic	*n* (%)	BBQ-Srb M (SD)	NRS M (SD)	ODI M (SD)	PCS M (SD)
Gender					
Male	44 (30.8%)	22.11 (5.81)	6.30 (1.53)	33.45 (19.35)	19.36 (11.99)
Female	99 (69.2%)	21.14 (6.05)	6.11 (1.28)	35.09 (17.57)	20.92 (11.02)
Age categories, years					
40–50	33 (23.1%)	21.67 (6.27)	6.44 (1.37)	37.39 (21.28)	23.24 (10.80)
51–60	65 (45.5%)	21.94 (5.89)	5.90 (1.15)	33.45 (13.86)	18.98 (11.12)
61–65	45 (31.5%)	20.56 (5.90)	6.36 (1.58)	34.18 (20.95)	20.49 (11.79)
BMI categories					
Underweight (≤18.4)	0 (0%)				
Normal weight (18.5–24.9)	29 (27.9%)	21.25 (6.77)	6.19 (1.04)	37.17 (14.87)	18.90 (9.43)
Overweight (25.0–29.9)	54 (51.9%)	21.63 (5.51)	6.11 (1.40)	32.63 (19.33)	20.65 (12.19)
Obesity (30.0–39.9)	21 (20.2%)	21.19 (6.18)	6.05 (1.24)	36.00 (16.85)	24.86 (12.29)
Severe obesity (≥40.0)	0 (0%)				
Marital status					
Married	101 (70.6%)	21.59 (6.48)	6.09 (1.32)	33.66 (17.62)	21.18 (10.82)
Single	12 (8.4%)	22.75 (6.48)	5.83 (1.34)	29.83 (14.66)	17.33 (10.86)
Divorced/separated	13 (9.1%)	19.00 (6.00)	6.77 (1.69)	43.08 (19.28)	21.77 (15.66)
Widowed	17 (11.9%)	21.47 (5.67)	6.41 (1.33)	36.94 (21.20)	17.24 (10.77)
Cohabitation					
Yes	57 (40.1%)	21.88 (5.73)	6.46 (1.38)	43.86 (17.95)	20.65 (11.48)
No	85 (59.9%)	21.12 (6.17)	5.99 (1.33)	28.31 (15.48)	20.45 (11.25)
Education					
Primary school	10 (7.0%)	18.40 (6.65)	6.90 (1.66)	32.60 (13.03)	19.80 (12.62)
High school	90 (62.9%)	21.53 (5.87)	6.04 (1.34)	35.96 (18.03)	20.51 (10.60)
College	18 (12.6%)	21.11 (4.98)	6.12 (1.22)	30.33 (19.17)	22.17 (10.89)
University degree	25 (17.5%)	22.56 (6.61)	6.38 (1.38)	33.52 (19.54)	19.20 (13.88)

M (SD)—mean (standard deviation); BBQ-Srb—Serbian version of the Back Beliefs Questionnaire; BBQ-Srb total score refers to the preliminary exploratory 8-item version excluding BBQ13; NRS—Numeric Rating Scale; ODI—Oswestry Disability Index; PCS—Pain Catastrophizing Scale; BMI—Body Mass Index.

**Table 2:** Inferential statistics for general participant characteristics.

Characteristic	BBQ-Srb, *p*-Value (η^2^)	NRS, *p*-Value (η^2^)	ODI, *p*-Value (η^2^)	PCS, *p*-Value (η^2^)
Gender	0.574 (0.006)	0.728 (0.000)	0.677 (0.015)	0.677 (0.009)
Age categories	0.574 (0.010)	0.669 (0.029)	0.728 (0.007)	0.669 (0.040)
BMI categories	0.921 (0.001)	0.933 (0.001)	0.677 (0.014)	0.669 (0.032)
Marital status	0.574 (0.020)	0.669 (0.032)	0.669 (0.038)	0.677 (0.033)
Cohabitation	0.574 (0.004)	0.669 (0.006)	<0.001 (0.226)	0.552 (0.025)
Education	0.574 (0.025)	0.669 (0.029)	0.728 (0.016)	0.933 (0.017)

*p*-values were adjusted using the Benjamini–Hochberg false discovery rate (FDR) correction for multiple comparisons. Effect sizes are presented as eta-squared (η^2^).

**Table 3:** Binary logistic regression models of the association between the BBQ-Srb total score and work absenteeism.

	BBQ Model	Full Model
BBQ-Srb (continuous)	1.034 (0.967–1.106)	1.150 (1.040–1.270) **
Gender (ref.: female)		
Male		0.950 (0.316–2.853)
Age (continuous)		0.941 (0.869–1.021)
ODI (continuous)		1.081 (1.033–1.132) **
PCS (continuous)		0.995 (0.948–1.045)
NRS (continuous)		0.946 (0.593–1.510)
Physical workload (ref.: physically demanding work)		
Mostly sitting		0.132 (0.021–0.805) *
Mostly standing/walking, but not carrying heavy loads		0.193 (0.031–1.200)
Walking a lot, climbing stairs, or lifting loads		0.325 (0.051–2.052)
Employment status (ref.: temporary/occasional employment)		
Regular employment		0.582 (0.068–4.954)

BBQ-Srb—Serbian version of the Back Beliefs Questionnaire; BBQ-Srb total score refers to the preliminary exploratory 8-item version excluding BBQ13; ODI—Oswestry Disability Index; PCS—Pain Catastrophizing Scale; NRS—Numeric Rating Scale; values are presented as Exp(B) with corresponding 95% confidence intervals; ** *p* < 0.01; * *p* < 0.05.

**Table 4:** Floor and ceiling effects for the original scored BBQ-Srb item set.

Scale/Item	First Administration						Second Administration					
	Floor (%)	Ceiling (%)	M	SD	Sk	Ku	Floor (%)	Ceiling (%)	M	SD	Sk	Ku
Original 9-item BBQ-Srb total score	0%	0%	23.14	6.25	0.14	−0.63	0%	0.7%	23.68	5.49	0.29	−0.26
BBQ1	17.5%	31.5%	3.28	1.49	−0.23	−1.36	12.6%	21.7%	3.24	1.32	−0.22	−1.08
BBQ2	21.0%	13.3%	2.62	1.29	0.53	−0.73	16.1%	13.3%	2.73	1.24	0.44	−0.69
BBQ3	38.5%	7.7%	2.20	1.22	0.81	−0.17	22.4%	7.0%	2.43	1.15	0.62	−0.31
BBQ6	24.5%	11.9%	2.53	1.28	0.57	−0.65	23.1%	10.5%	2.54	1.24	0.54	−0.59
BBQ8	7.7%	40.6%	3.73	1.29	−0.64	−0.71	5.6%	35.7%	3.71	1.23	−0.57	−0.73
BBQ10	19.6%	11.9%	2.82	1.25	0.11	−0.82	15.4%	9.1%	2.83	1.16	0.10	−0.64
BBQ12	32.9%	7.7%	2.28	1.20	0.73	−0.21	30.1%	7.0%	2.33	1.19	0.66	−0.35
BBQ13	56.6%	2.1%	1.71	0.98	1.40	1.51	50.3%	1.4%	1.83	1.01	1.04	0.27
BBQ14	42.7%	4.9%	1.99	1.12	1.13	0.63	39.9%	4.2%	2.05	1.12	0.95	0.21

M—mean; SD—standard deviation; Sk—skewness; Ku—kurtosis; floor and ceiling values are expressed as percentages; the floor and ceiling effect analysis presented in this table refers to the original 9-item BBQ-Srb scored version; item-level descriptive statistics are presented for all original scored BBQ-Srb items, including BBQ13.

**Table 5:** Reliability and structural validity of the BBQ-Srb.

Items	I-TC	CA-iid	ICC (95% CI)	EFA Loading	h^2^	CFA β	*p*
				(32.5%) ^#^			
BBQ1	0.373	0.708	0.87 (0.82–0.90)	0.382	0.146	0.333	ref.
BBQ2	0.559	0.67	0.85 (0.80–0.89)	0.707	0.500	0.722	<0.001
BBQ3	0.318	0.715	0.68 (0.57–0.76)	0.344	0.118	0.322	0.01
BBQ6	0.461	0.689	0.91 (0.88–0.94)	0.613	0.376	0.654	<0.001
BBQ8	0.465	0.688	0.92 (0.89–0.94)	0.574	0.329	0.578	<0.001
BBQ10	0.313	0.716	0.86 (0.81–0.90)	0.346	0.120	0.321	0.01
BBQ12	0.355	0.708	0.83 (0.73–0.85)	0.398	0.158	0.373	0.005
BBQ13	0.213	0.728	0.80 (0.74–0.85)	0.108	0.043	NA	NA
BBQ14	0.547	0.677	0.91 (0.87–0.93)	0.690	0.476	0.699	<0.001
Cronbach α			0.728 (95% CI 0.655–0.790)				
ICC total score (95% CI)			0.916 (0.883–0.940)				
AVE			0.28				
McDonald’s ω			0.735				
SEM			1.69				
SDC			4.68				
CR			0.74				
χ^2^ (df)			41.82 (20)				
χ^2^/df			2.09				
CFI			0.889				
TLI			0.844				
RMSEA (90% CI)			0.088 (0.050–0.125)				
SRMR			0.143				

BBQ item labels refer to the corresponding items of the BBQ-Srb; I-TC—item-total correlation; CA-iid—Cronbach’s alpha if item deleted; the 95% confidence interval for Cronbach’s alpha was calculated using Feldt’s method; h^2^—item communality, representing the proportion of item variance explained by the extracted factor(s); ICC—intraclass correlation coefficient calculated using a two-way mixed-effects model, absolute agreement definition, and single-measure coefficients; SEM—Standard Error of Measurement; SDC—Smallest Detectable Change; ^#^—Percentage of Variance; principal axis factoring with direct oblimin rotation; AVE—Average Variance Extracted; CR—Composite reliability; EFA—exploratory factor analysis; CFA (β)—standardized factor loading from confirmatory factor analysis; NA—not applicable; ref.—reference category; χ^2^—Chi-square test of model fit; df—degrees of freedom; CFI—Comparative Fit Index; TLI—Tucker–Lewis Index; RMSEA—Root Mean Square Error of Approximation; SRMR—Standardized Root Mean Square Residual. KMO = 0.737; Bartlett’s test of sphericity: χ^2^ = 232.786; df = 36; *p* < 0.001. BBQ13 is presented only to show its poor item-level performance. Cronbach’s α, ICC, SEM, SDC, construct-validity analyses, and regression analyses are based on the preliminary exploratory 8-item BBQ-Srb total score excluding BBQ13. The initial 9-item scored BBQ-Srb version had Cronbach’s α = 0.725.

**Table 6:** Hypothesis testing for construct validity of the BBQ-Srb.

N	Hypothesis Testing	Estimated Correlation, *p* Value	Hypothesis Supported
1	BBQ-Srb total score was expected to show a weak to moderate negative correlation with PCS scores.	r = −0.403, *p* < 0.001	Yes
2	BBQ-Srb total score was expected to show a weak to moderate negative correlation with ODI scores.	r = −0.284, *p* = 0.001	Yes
3	BBQ-Srb total score was expected to show a weak to moderate negative correlation with NRS scores.	r = −0.256, *p* = 0.002	Yes
4	A lower BBQ-Srb total score was expected to be associated with greater odds of work absenteeism.	Adjusted OR = 1.150, 95% CI: 1.040–1.270, *p* < 0.01	No; the association was statistically significant, but the direction was opposite to the predefined hypothesis.

BBQ-Srb—Serbian version of the Back Beliefs Questionnaire; BBQ-Srb total score refers to the preliminary exploratory 8-item version excluding BBQ13; Pearson’s correlation coefficients were used for hypotheses 1–3. Binary logistic regression was used for hypothesis 4, with sex, age, ODI, PCS, NRS, employment status, and physical workload entered as covariates. OR—odds ratio; CI—confidence interval.

## Data Availability

The datasets generated and/or analyzed during the current study are available from the corresponding author upon reasonable request. The data are not publicly available due to privacy and ethical restrictions.

## Supplementary Materials

The following supporting information can be downloaded at: https://www.mdpi.com/article/10.3390/medicina62061174/s1, Table S1. Occupational and pain-related characteristics and descriptive scale scores; Table S2. Inferential statistics for occupational and pain-related characteristics.

## Abbreviations

The following abbreviations are used in this manuscript:

LBP	Low Back Pain
BBQ	Back Beliefs Questionnaire
BBQ-Srb	Serbian version of the Back Beliefs Questionnaire
ODI	Oswestry Disability Index
PCS	Pain Catastrophizing Scale
NRS	Numeric Rating Scale
EFA	Exploratory Factor Analysis
CFA	Confirmatory Factor Analysis
ICC	Intraclass Correlation Coefficient
BMI	Body Mass Index
AVE	Average Variance Extracted
SEM	Standard Error of Measurement
SDC	Smallest Detectable Change
KMO	Kaiser–Meyer–Olkin measure of sampling adequacy
ANOVA	Analysis of Variance
FDR	False Discovery Rate
