# Supplementary material for: Cross-Cultural Adaptation and Psychometric Validation of the Serbian Version of the Back Beliefs Questionnaire in Patients with Chronic Low Back Pain
Source: Medicina (Kaunas). 2026 Jun 17;62(6):1174. doi: 10.3390/medicina62061174 (PMC13304279; doi:10.3390/medicina62061174)
Supplement: Supplementary file 1 [file medicina-62-01174-s001.zip › medicina-4271534-supplementary.pdf]

Table S1. Occupational and pain-related characteristics and descriptive scale scores.

Characteristic	n (%)	BBQ-Srb M (SD)	NRS M (SD)	ODI M (SD)	PCS M (SD)
Occupation*					
Professionals/experts	14 (9.8%)	24.64 (6.17)	6.31 (1.32)	31.71 (19.87)	18.93 (11.09)
Associate professionals/technicians	30 (21.0%)	20.73 (6.41)	5.83 (1.21)	30.40 (11.84)	19.53 (13.40)
Administrative staff	22 (15.4%)	20.14 (5.49)	6.52 (1.63)	37.45 (21.56)	20.14 (10.78)
Service/sales workers	38 (26.6%)	21.05 (6.71)	6.00 (1.25)	37.00 (19.02)	22.61 (11.54)
Other	39 (27.3%)	21.95 (4.77)	6.36 (1.42)	34.87 (18.53)	19.74 (9.92)
Employment status*					
Permanent	96 (67.1%)	21.76 (6.27)	6.09 (1.25)	34.54 (17.82)	21.05 (11.63)
Temporary	8 (5.6%)	20.25 (5.28)	6.50 (1.51)	35.00 (16.70)	20.50 (11.83)
Retired	9 (6.3%)	23.11 (4.70)	6.22 (1.92)	36.89 (15.72)	19.67 (8.82)
Housewife	26 (18.2%)	19.77 (5.18)	6.31 (1.59)	34.31 (21.63)	19.08 (11.68)
Unable to work	4 (2.8%)	23.25 (7.09)	6.50 (1.00)	31.50 (13.70)	16.25 (6.80)
Job alignment*					
Yes	77 (54.2%)	21.19 (5.88)	6.20 (1.42)	33.17 (17.52)	19.75 (11.90)
No	40 (28.2%)	21.53 (6.75)	6.00 (1.38)	33.90 (17.34)	22.13 (11.09)
Partially	25 (17.6%)	22.08 (5.16)	6.32 (1.18)	38.16 (18.97)	19.76 (10.09)
Workload*					
Mostly sitting	37 (25.9%)	23.00 (6.08)	6.00 (1.29)	29.24 (13.74)	17.05 (11.52)
Mostly standing/walking, no heavy loads	48 (33.6%)	20.75 (5.47)	6.38 (1.62)	34.75 (21.07)	19.50 (9.87)
Mostly standing/walking, moderate loads	38 (26.6%)	21.50 (6.23)	6.16 (1.17)	40.84 (18.72)	22.03 (11.82)
Heavy physical work, heavy loads	20 (14.0%)	20.10 (6.24)	6.00 (1.17)	32.20 (12.86)	25.95 (11.40)
Back pain status					
At rest	7 (4.9%)	22.71 (6.68)	6.14 (1.46)	22.00 (6.63)	20.71 (11.25)
During work	42 (29.6%)	23.02 (6.53)	5.66 (1.04)	27.76 (15.74)	15.76 (9.58)
At rest and during work	93 (65.5%)	20.55 (5.52)	6.41 (1.44)	38.62 (18.49)	22.63 (11.51)
Pain location					
Lower back only	31 (21.7%)	22.61 (5.79)	5.53 (1.01)	26.26 (12.56)	17.84 (9.88)
Radiates occasionally to leg	73 (51.0%)	21.38 (5.86)	6.30 (1.40)	35.78 (18.30)	19.48 (11.02)
Radiates constantly to leg	39 (27.3%)	20.62 (6.31)	6.44 (1.41)	38.97 (19.60)	24.31 (12.18)
Employee absenteeism*					
No	35 (33.7%)	20.64 (6.26)	5.88 (1.37)	25.31 (14.38)	20.26 (11.90)
Yes	69 (66.3%)	21.95 (5.75)	6.24 (1.21)	39.28 (17.38)	21.39 (11.50)
Duration of absenteeism*					
None	35 (33.7%)	20.64 (6.26)	5.88 (1.37)	25.31 (14.38)	20.26 (11.90)

A few days a year	39 (37.5%)	23.15 (4.75)	6.14 (1.23)	39.33 (17.26)	18.33 (9.02)
A few weeks a year	22 (21.2%)	21.37 (6.53)	6.45 (1.10)	39.18 (18.64)	24.77 (14.09)
A few months a year	8 (7.7%)	18.00 (6.27)	6.13 (1.46)	39.25 (16.63)	27.00 (11.12)
Frequency of absenteeism*					
Once a year	9 (8.7%)	22.29 (5.88)	5.44 (0.73)	31.11 (15.91)	21.67 (7.55)
Twice a year	28 (26.9%)	23.00 (5.65)	5.85 (0.97)	28.29 (15.54)	20.93 (10.63)
Three or more times a year	67 (64.4%)	20.78 (6.04)	6.32 (1.38)	37.67 (18.14)	20.96 (12.51)
All respondents	143 (100%)	21.44 (5.97)	6.17 (1.36)	34.58 (18.08)	20.44 (11.31)

*The calculation was performed on a sample of employed respondents (N = 104). M (SD) – Mean (Standard Deviation); BBQ-Srb – Serbian version of the Back Beliefs Questionnaire; BBQ-Srb total score refers to the preliminary exploratory 8-item version excluding BBQ13; NRS – Numeric Rating Scale; ODI – Oswestry Disability Index; PCS – Pain Catastrophizing Scale; ANOVA p-values were adjusted using the Benjamini–Hochberg false discovery rate (FDR) correction for multiple comparisons. Effect sizes are presented as Eta-squared (η^2).

Table S2. Inferential statistics for occupational and pain-related characteristics.

Characteristic	BBQ-Srb p-value (η^2)	NRS p-value (η^2)	ODI p-value (η^2)	PCS p-value (η^2)
Occupation*	0.366 (0.042)	0.556 (0.047)	0.717 (0.022)	0.834 (0.031)
Employment status*	0.509 (0.026)	0.924 (0.008)	0.992 (0.000)	0.924 (0.000)
Job alignment*	0.812 (0.003)	0.768 (0.011)	0.677 (0.003)	0.717 (0.021)
Workload*	0.366 (0.029)	0.735 (0.001)	0.137 (0.076)	0.117 (0.073)
Back pain status	0.306 (0.038)	0.067 (0.037)	0.018 (0.102)	0.036 (0.086)
Pain location	0.490 (0.014)	0.067 (0.098)	0.065 (0.068)	0.126 (0.060)
Work absenteeism*	0.366 (0.012)	0.388 (0.018)	0.018 (0.141)	0.768 (0.002)
Duration of absenteeism*	0.306 (0.058)	0.662 (0.027)	0.024 (0.141)	0.206 (0.065)
Frequency of absenteeism*	0.366 (0.026)	0.206 (0.054)	0.147 (0.058)	0.992 (0.000)

*The calculation was performed on a sample of employed respondents (N = 104). BBQ-Srb – Serbian version of the Back Beliefs Questionnaire; BBQ-Srb total score refers to the preliminary exploratory 8-item version excluding BBQ13; NRS – Numeric Rating Scale; ODI – Oswestry Disability Index; PCS – Pain Catastrophizing Scale; analysis of variance p-values were adjusted using the Benjamini–Hochberg false discovery rate (FDR) correction for multiple comparisons. Effect sizes are presented as eta-squared (η^2).
